# Triage of Women with Low-Grade Cervical Lesions - HPV mRNA Testing versus Repeat Cytology

**DOI:** 10.1371/journal.pone.0024083

**Published:** 2011-08-30

**Authors:** Sveinung Wergeland Sørbye, Marc Arbyn, Silje Fismen, Tore Jarl Gutteberg, Elin Synnøve Mortensen

**Affiliations:** 1 Department of Clinical Pathology, The University Hospital of North Norway, Tromsø, Norway; 2 Belgian Cancer Center/Unit of Cancer Epidemiology, Scientific Institute of Public Health, Brussels, Belgium; 3 Department of Microbiology and Infection Control, The University Hospital of North Norway, Tromsø, Norway; 4 Department of Medical Biology, Faculty of Health Sciences, University of Tromsø, Tromsø, Norway; Karolinska Institutet, Sweden

## Abstract

**Background:**

In Norway, women with low-grade squamous intraepithelial lesions (LSIL) are followed up after six months in order to decide whether they should undergo further follow-up or be referred back to the screening interval of three years. A high specificity and positive predictive value (PPV) of the triage test is important to avoid unnecessary diagnostic and therapeutic procedures.

**Materials and Methods:**

At the University Hospital of North Norway, repeat cytology and the HPV mRNA test PreTect HPV-Proofer, detecting E6/E7 mRNA from HPV types 16, 18, 31, 33 and 45, are used in triage of women with ASC-US and LSIL. In this study, women with LSIL cytology in the period 2005–2008 were included (n = 522). Two triage methods were evaluated in two separate groups: repeat cytology only (n = 225) and HPV mRNA testing in addition to repeat cytology (n = 297). Histologically confirmed cervical intraepithelial neoplasia of grade 2 or worse (CIN2+) was used as the study endpoint.

**Results:**

Of 522 women with LSIL, 207 had biopsies and 125 of them had CIN2+. The sensitivity and specificity of repeat cytology (ASC-US or worse) were 85.7% (95% confidence interval (CI): 72.1, 92.2) and 54.4 % (95% CI: 46.9, 61.9), respectively. The sensitivity and specificity of the HPV mRNA test were 94.2% (95% CI: 88.7, 99.7) and 86.0% (95% CI: 81.5, 90.5), respectively. The PPV of repeat cytology was 38.4% (95% CI: 29.9, 46.9) compared to 67.0% (95% CI: 57.7, 76.4) of the HPV mRNA test.

**Conclusion:**

HPV mRNA testing was more sensitive and specific than repeat cytology in triage of women with LSIL cytology. In addition, the HPV mRNA test showed higher PPV. These data indicate that the HPV mRNA test is a better triage test for women with LSIL than repeat cytology.

## Introduction

Infection Human Papillomavirus (HPV) is a necessary cause of cervical cancer. In Norway, the cervical cancer screening program is based on cytology. The rationale of cervical cancer cytological screening is to identify and treat high-grade cervical intraepithelial neoplasia (CIN) (precancerous lesions) in order to prevent its progression to invasive cancer. Women with high-grade intraepithelial squamous lesions (HSIL) detected at cytological screening are referred to further assessment with colposcopy and biopsy[1], [2]. Women with minor cervical lesions have a small but significantly increased risk of developing cervical cancer compared to women with normal smears. The purpose of triage is to identify women needing further follow-up by colposcopy and biopsy to detect high grade cervical dysplasia or cancer (CIN2+). In case of atypical squamous cell of undetermined significance (ASC-US), it is now widely recognized that triage with an HPV DNA test is more sensitive, but less specific than repeat cytology[3], [4]. For low-grade squamous intraepithelial lesions (LSIL) however, recommendations regarding the best triage method are conflicting and vary from repeat cytology, HPV testing or direct referral to colposcopy[5], [6]. Meta-analyses indicate that triage of LSIL with a high-risk HPV DNA test is not more sensitive and substantially less specific than repeat cytology[3], [4]. In some studies, HPV-based triage of LSIL in women older than 30–35 years has been suggested[7], [8], but other studies have not confirmed this[9], [10]. Defining the best strategy to triage LSIL lesions is therefore identified as a priority for research.

In Norway, HPV testing has been included in triage since 2005. If the cytological findings are defined as ASC-US or LSIL, women are referred to repeat cytology and HPV test after 6 months. If the repeat cytology reveals ASC-US or LSIL and the HPV test is positive, the patient is referred to colposcopy and biopsy. Women with normal Pap-smear at repeat cytology, but with a positive HPV result, are tested for HPV persistence and if positive, referred to colposcopy. Women with cytological findings defined as high grade squamous intraepithelial lesion (HSIL) or atypical squamous cell cannot exclude HSIL (ASC-H) at triage are referred to colposcopy and biopsy independent of the HPV result. The flow-chart showing the guidelines for follow-up of minor cytological lesions is presented in Sørbye et al., 2010b[11].

In the routine diagnostic practice at the University Hospital of North Norway (UNN), the HPV E6/E7 mRNA test PreTect HPV-Proofer is used in the triage of women with ASC-US or LSIL. In the current study, the accuracy of two triage methods to predict underlying or incipient CIN2+ was assessed, in women with a first result of LSIL. The first group was triaged with repeat cytology only whereas the second group was triaged with repeat cytology and HPV mRNA testing with PreTect HPV-Proofer.

## Materials and Methods

### Ethics Statement

The Regional Committee for Research Ethics (REK Sør-Øst C) has approved the study. Written consent from the patients for their information to be stored in the hospital database and used for research was not needed because the data were obtained and analyzed anonymously.

In the routine diagnostic practice at the University Hospital of North Norway, the HPV E6/E7 mRNA test PreTect HPV-Proofer (detecting E6/E7 mRNA from the HPV types 16, 18, 31, 33 and 45; NorChip AS, Norway) is used in the triage of women with a Pap-smear showing ASC-US or LSIL. The Department of Clinical Pathology receives cervical smears from the population of Troms and Finnmark County. Approximately 23 000 cervical smears are analysed each year. In 2005–2008, smears from 57 560 women aged 25–69 years were processed. During that period, 849 women (1.5%) had a cytological result of LSIL.

Some patients were excluded from the study population. First of all, women with previous treatment of CIN2+ were excluded (n = 41). Also, women with a previous abnormal smear were excluded (n = 209), since they are managed differently and because they are at higher risk of having a high-grade lesion compared to women with a first LSIL result. According to the national guidelines, women with LSIL should be followed up with two normal Pap-smears at least 12 months apart or alternatively, with one normal liquid based cytology (LBC) and a negative HPV-test, before returning to routine screening at an interval of three years[11]. Due to this, women with only one normal Pap-smear during follow-up (n = 23) were excluded from the study. Lastly, women with one or several abnormal Pap-smears and/or a positive HPV-test at follow-up but without biopsy were excluded (n = 54).

**Figure 1:**
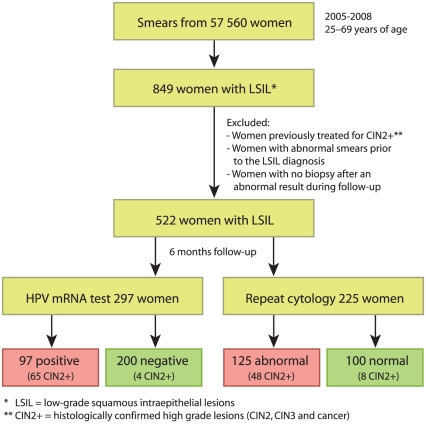
Flow chart showing the two study populations.

Considering the criteria above, 522 of 849 (61.5%) women with LSIL were included in the study. Among these 522 women, an LBC follow-up sample was taken for 297 women (56.9%), allowing for additional HPV mRNA testing. From the other 225 women (43.1%), a conventional Pap-smear was received not allowing for HPV analysis (see Figure 1 and Table 1).

**Table 1:** Total number of women with LSIL 1) and with histologically confirmed CIN2+ 2) by cytological result of repeat cytology.

Repeat cytology	Number of women	Number of women referred to biopsy	Number of women with CIN2+	PPV (%)	95% CI
Normal	221	35	12	5.4	2.4, 8.4
ASC-US	90	29	15	16.7	9.0, 24.4
LSIL	154	87	54	35.1	27.5, 42.6
ASC-H	30	29	21	70.0	53.6, 86.4
HSIL	27	27	23	85.2	71.8, 98.6
Total	522	207	125	23.9	20.3, 27.6

1) LSIL  =  low-grade squamous intraepithelial lesions.

2) CIN2+  =  CIN2, CIN3 and cancer.

In LBC, cells were extracted from the ThinPrep® 2000 (Cytyc Corporation, Marlborough, MA, USA) for cytological examination. Of the remnant liquid with cervical cells, DNA/RNA was isolated from 5 ml sample and eluted in 50 µl elution buffer and analysed with PreTect HPV-Proofer. The mRNA testing was performed according to the manufacturer's instructions (NorChip AS) and in accordance with national guidelines for HPV testing[11].

Cytological and histological diagnoses were obtained from the diagnostic database (SymPathy) at the Department of Clinical Pathology, UNN. Biopsies were evaluated by experienced pathologists and histological results were reported using CIN terminology[12]. In Norway, the threshold for treatment by conisation is CIN2. Women with benign or CIN1 histology are advised to be followed up with a new Pap-smear and HPV testing after 6–12 months. Biopsies with uncertain cellular changes are analyzed with p16(INK4a) immunostaining (CINtec® Histology, MTM, Heidelberg, Germany) in order to detect occult CIN lesions.

Outcome assessment was based on the histological result of biopsies, where CIN2+ was considered as the target disease and CIN1 and CIN0 (no CIN) were considered as absence of disease. Moreover, women with a complete negative follow-up (two consecutive negative Pap-smears or double negative LBC/mRNA HPV result) were assumed to be free of disease. The clinical sensitivity, specificity, positive predictive value (PPV) and negative predictive value (NPV) were calculated in 2×2 tables for cytology (with cut-off ASC-US+ and ASC-H+, respectively), for the HPV-test alone, and HPV-test in combinations with cytology (with cut-off ASC-US+ and ASC-H+). Pearson's Chi square was used to assesses differences in the accuracy of repeat cytology alone, HPV-testing alone and HPV-testing in combination with cytology.

The statistical computations were performed with the software R version 2.9.0 (2009-04-17), http://www.r-project.org/. P-value <0.05 was considered statistically significant.

## Results

In Table 1, outcomes by triage cytology 6 months after the index LSIL diagnosis are presented for all the 522 women included. Furthermore, the PPVs for the different cytological triage results are presented.

Of the 225 women in the “repeat cytology only” group (Tables 2 and 3), 85 women were referred to biopsy; 56 women (24.9%) had CIN2+; 29 women had CIN1 or CIN0. Hundred and forty women were not referred to histology due to two normal Pap-smears 6 months apart or alternatively one normal LBC and a negative HPV result which was considered as equivalent to “no disease”. Altogether, 169 women showed a negative outcome (29 with a negative biopsy and 140 with negative follow-up with cytology and HPV testing). The accuracy for CIN2+ of triage with cytology with cut-off ASC-US+ is presented in Table 2. The estimates of sensitivity, specificity, PPV and NPV were 85.7%, 54.4%, 38.4% and 92.0%, respectively. With cut-off ASC-H+, the sensitivity was 33.9 %, the specificity 97.6%, the PPV 82.6% and the NPV 81.7% (Table 3).

**Table 2:** Outcome for the 225 women triaged only with repeat cytology when cut-off is ASC-US+ 1).

Cytological findings	Outcome				%	95% CI
	CIN2+ 3)	<CIN2 4)	Total 5)	Sensitivity	85.7	72.1, 92.2
ASC-US+	48	77	125	Specificity	54.4	46.9, 61.9
NILM 2)	8	92	100	PPV	38.4	29.9, 46.9
Total	56	169	225	NPV	92.0	86.7, 97.3

1) ASC-US  =  atypical squamous cell of undetermined significance, ASC-US+  =  ASC-US, LSIL, ASC-H and HSIL.

2) NILM  =  Negative for intraepithelial lesion or malignancy.

3) Prevalence CIN2+ 24.9% (95% CI, 19.2, 30.5).

4) <CIN2 includes women with a histological diagnosis of CIN1 or CIN0 (n = 29) and women referred back to screening because of two normal follow-up smears 12 months apart (n = 140).

5) Chi square  =  25.9, p<0.001.

**Table 3:** Outcome for the 225 women triaged only with repeat cytology when the cut-off is ASC-H+.

Cytological findings	Outcome				%	95% CI
	CIN2+	<CIN2 2)	Total 3)	Sensitivity	33.9	21.5, 46.3
ASC-H+1)	19	4	23	Specificity	97.6	95.3, 99.9
<ASC-H	37	165	202	PPV	82.6	67.1, 98.1
Total	56	169	225	NPV	81.7	76.4, 87.0

1) ASC-H  =  atypical squamous cell cannot exclude HSIL, ASC-H+  =  ASC-H and HSIL.

2) <CIN2 includes women with a histological diagnosis of CIN1 or CIN0 (n = 29) and women referred back to screening because of two normal follow-up smears 12 months apart (n = 140).

3) Chi square  =  42.3, p<0.001.

Of the 297 women with HPV mRNA test (Tables 4 Table 5 Table 6), 122 women were referred to biopsy; 69 women (23.2%) had CIN2+; 53 women had CIN1 or CIN0. Altogether, 228 had a negative outcome (53 with a negative biopsy and 175 with negative follow-up with cytology and HPV testing). The accuracy parameters for triage with HPV mRNA only are shown in Table 4. The estimates of sensitivity, specificity, PPV and NPV were 94.2%, 86.0%, 67.0%, and 98.0%, respectively. HPV mRNA test in combination with cytology with cut-off ASC-US+ has a specificity of 47.4% and a PPV of 36.5% (Table 5). Sensitivity and NPV were not evaluated for the combination since by design they must be 100%. The HPV mRNA test in combination with cytology with cut-off ASC-H+ had sensitivity 98.6%, specificity 83.8% and PPV 64.8% (Table 6).

**Table 4:** Outcome for the 297 women triaged only with HPV mRNA.

HPV mRNA result	Outcome			%	%	95% CI
	CIN2+ 1)	<CIN2 2)	Total 3)	Sensitivity	94.2	88.7, 99.7
Positive	65	32	97	Specificity	86.0	81.5, 90.5
Negative	4	196	200	PPV	67.0	57.7, 76.4
Total	69	228	297	NPV	98.0	96.1, 99.9

Number of women found with high grade lesion (CIN2+) and number of women referred back to the screening program (<CIN2).

1) Prevalence CIN2+ 23.2% (95% CI, 18.4, 28.0).

2) <CIN2 includes women with a histological diagnosis of CIN1 or CIN0 (n = 53) and women referred back to screening because of two normal follow-up smears 12 months apart or one normal LBC and a negative HPV result (n = 175).

3) Chi square  =  151.2, p<0.001.

**Table 5:** Outcome for the 297 women triaged with HPV mRNA when combined with cytology with cut-off ASC-US+.

Combination result	Outcome				%	95% CI
	CIN2+	<CIN2 1)	Total 2)			
Positive	69	120	189	Specificity	47.4	49.9, 53.8
Negative	0	108	108	PPV	36.5	56.6, 70.4
Total	69	228	297			

1) <CIN2 includes women with a histological diagnosis of CIN1 or CIN0 (n = 53) and women referred back to screening because of two normal follow-up smears 12 months apart or one normal LBC and a negative HPV result (n = 175).

2) Chi square  =  49.3, p<0.001.

**Table 6:** Outcome for the 297 women triaged with HPV mRNA when combined with cytology with cut-off ASC-H+.

Combination result	Outcome				%	95% CI
	CIN2+	<CIN2 1)	Total 2)	Sensitivity	98.6	95.7, 100.0
Positive	68	37	105	Specificity	83.8	79.0, 88.6
Negative	1	191	192	PPV	64.8	55.6, 73.9
Total	69	228	297	NPV	99.5	98.5, 100.0

1) <CIN2 includes women with a histological diagnosis of CIN1 or CIN0 (n = 53) and women referred back to screening because of two normal follow-up smears 12 months apart or one normal LBC and a negative HPV result (n = 175).

2) Chi square  =  153.5, p<0.001.

In total, 7 of the 522 women (1.3%) with LSIL were diagnosed with cervical cancer in the follow-up. Two were detected in the “repeat cytology only” group and five were detected in the HPV group. The five women with cervical cancer in the HPV group were all HPV mRNA positive for HPV type 16 (data not shown).

## Discussion

The main cause of invasive cervical cancer is the deregulated and persistent production of HPV E6 and E7 oncoproteins[13]. Hence, HPV E6/E7 mRNA is a rational target for detecting HPV infections leading to cellular transformation. PreTect HPV-Proofer detects E6/E7 mRNA of the five main high-risk HPV types 16, 18, 31, 33, and 45. Due to the higher clinical specificity and PPV of this method compared to other HPV tests[14]–[18], this was the method of choice at our hospital when HPV testing was introduced in triage of women with minor cytological lesions in Norway.

When summarizing results from all women included in the study (Table 1), 125 of 522 women (23.9%) had CIN2+ confirmed by biopsy. Further, the PPV of ASC-US and LSIL in repeat cytology after an LSIL diagnosis was relatively low, i.e., the chance of having disease even with a 6 months persistent low-grade diagnosis was concordantly relatively low. Previously, before HPV testing was included in clinical practice, all women with persistent ASC-US/LSIL were referred to follow-up after one year. In current practice, this is still the case for women with repeated ASC-US/LSIL and a negative HPV mRNA result. Women with a positive HPV result however, are referred directly to colposcopy and biopsy[11]. A high PPV of the HPV test is therefore important in order to avoid unnecessary follow-up, both with regard to follow-up costs to the health care system and with regard to unnecessary psychological stress among patients. In addition, it is known that HPV tests identify lesions not found by cytology and thereby contribute in increasing the overall clinical sensitivity of the screening program[3], [4], [19]–[23].

It is also important to have in mind that in clinical practice, the cytological diagnosis is commonly influenced by the HPV result. We see a tendency that a preliminary diagnosis of for example normal or ASC-US is upgraded when a positive HPV mRNA result is acknowledged. As a result, the clinical sensitivity of cytology as such will be over-estimated. In order to get two groups for comparison, a study with two separate arms was conducted. One arm with repeat cytology only and one arm with both repeat cytology and HPV test. National guidelines recommend that a woman with LSIL should be followed up with repeat cytology and an HPV test. The HPV-test however is not always performed due to use of traditional Pap-smears instead of LBC, a method not allowing for HPV testing. This made it possible to get a separate arm with repeat cytology only. The HPV test result is objective and is not dependent on the cytological diagnosis. Women referred to histology based on the cytological result alone (i.e., having a negative HPV result) made it possible to calculate clinical properties also including HPV negative results.

Until 2006, only conventional Pap-smears were used. When HPV-testing was introduced, liquid based cytology was recommended, but still almost half of the samples for repeat cytology are conventional Pap-smears. This can partly be explained by the fact that doctors are not properly informed about the possibility to perform concomitant HPV-test using LBC. Thus, the physician, and not the patient, is the reason for HPV-testing not being performed. Based on the above, one limitation of the study is the absence of formal randomization, which can result in imbalanced groups. Still, one argument about comparability is the similar age and similar prevalences of CIN2+. The age was similar of the two groups with overlapping confidence intervals. In the group with repeat cytology only the average age was 36.7 years (95 % CI: 35.6, 37.8); the median age was 34 years. In the HPV group the average age was 37.8 years (95 % CI: 36.5, 39.1); the median age was 36 years. The prevalence of CIN2+ was similar in the two groups (24.9% and 23.2%, respectively).

Another limitation of the study is a relatively young study population due to the exclusion of women with previous abnormal smears or HPV tests and the population will therefore not reflect a general LSIL population.

Referral to biopsy in the HPV-category is based on both the cytology and the HPV result and therefore does not represent the true number of women referred to biopsy based on HPV testing alone. For example, women with HSIL and ASC-H are always referred to biopsy independent of the HPV result. An argument against differential intensity of verification in the repeat cytology only group and the HPV group is the similar biopsy referral rate. The referral rate for biopsy was similar in the two groups (37.8% and 41.1%, respectively).

With regard to repeat cytology, statistical parameters were calculated both at cutoff ASC-US+ and ASC-H+. The reason for this is different follow up in the two groups. Women with ASC-H+ are referred to further assessment with colposcopy and biopsy. Women with ASC-US and LSIL are followed up with cytology and HPV-test. Repeat cytology with cut-off ASC-US+ has a high sensitivity (85.7%) for CIN2+, but only a moderate specificity (54.4%). In contrast, repeat cytology with cut-off ASC-H+ has a low sensitivity (33.9%), but a high specificity (97.6%). The combination of HPV mRNA testing and cytology with cut-off ASC-H+ has both high sensitivity (98.6%) and high specificity (83.8%). For comparison, the average specificity of the Hybrid Capture 2 HPV DNA test in triage of LSIL is 28.6% (95% CI: 22.2, 35.0)[24] and generally, high HPV positivity among women with LSIL explains why HPV DNA testing is not recommended in triage of women with LSIL[9].

In the present study, the HPV mRNA test shows a specificity of 86.0 % (95% CI: 81.5, 90.5) and has previously been shown to have a low positivity rate in woman with LSIL[25]. Also, previous data on HPV mRNA testing in the triage of ASC-US and LSIL[11], [25] show that the HPV mRNA test, due to a high specificity and positive predictive value (PPV), is valuable in the triage of women with minor cytological cervical lesions as it gives important information in terms of further follow-up. In fact, when the HPV result is positive, the data suggest direct treatment for women above 40 years of age or for women with a concurrent cytological HSIL diagnosis, contributing to better clinical safety for these women[11].

In the present study, the HPV mRNA test is more sensitive and significantly more specific than repeat cytology in triage of women with LSIL cytology. In addition, the HPV mRNA test has a significantly higher PPV and NPV than cytology alone. A high Chi square indicates high accuracy for the triage test. The HPV mRNA test alone and HPV mRNA test in combination with ASC-H+ had high Chi square values. These data indicate that the HPV mRNA test is a better triage test for women with LSIL than repeat cytology. The use of HPV mRNA test can reduce the time from abnormal smear to diagnosis and treatment for CIN2+. Further studies are needed in order to reveal whether HPV mRNA testing and E6/E7 detection may replace repeat cytology in follow-up of women with cytological low-grade lesions.
